# An Integrated Approach to Design and Develop High-Performance Polymer-Composite Thermal Interface Material

**DOI:** 10.3390/polym13050807

**Published:** 2021-03-06

**Authors:** Syed Sohail Akhtar

**Affiliations:** 1 Mechanical Engineering Department, King Fahd University of Petroleum and Minerals (KFUPM), Dhahran 31261, Saudi Arabia; ssakhtar@kfupm.edu.sa; 2 Interdisciplinary Research Center for Intelligent Manufacturing and Robotics, KFUPM, Dhahran 31261, Saudi Arabia; 3 Center of Excellence in Nanotechnology, KFUPM, Dhahran 31261, Saudi Arabia

**Keywords:** interface, design, composites, polymer, thermal resistance

## Abstract

A computational framework based on novel differential effective medium approximation and mean-field homogenization is used to design high-performance filler-laden polymer thermal interface materials (TIMs). The proposed design strategy has the capability to handle non-dilute filler concentration in the polymer matrix. The effective thermal conductivity of intended thermal interface composites can be tailored in a wide range by varying filler attributes such as size, aspect ratio, orientation, as well as filler–matrix interface with an upper limit imposed by the shear modulus. Serval potential polymers and fillers are considered at the design stage. High-density polyethylene (HDPE) and thermoplastic polyurethane (TPU) with a non-dilute concentration (~60 vol%) of ceramic fillers exhibit high thermal conductivity (4–5 W m^−1^ K^−1^) without compromising the high compliance of TIMs. The predicted thermal conductivity and coefficient of thermal expansion are in excellent agreement with measured data of various binary composite systems considering HDPE, TPU, and polypropylene (PP) loaded with Al_2_O_3_ and AlN fillers in varying sizes, shapes, and concentrations, prepared via the melt-mixing and compression-molding route. The model also validates that manipulating filler alignment and aspect ratio can significantly contribute to making heat-conducting networks in composites, which results in ultra-high thermal conductivity.

## 1. Introduction

**Figure 1:**
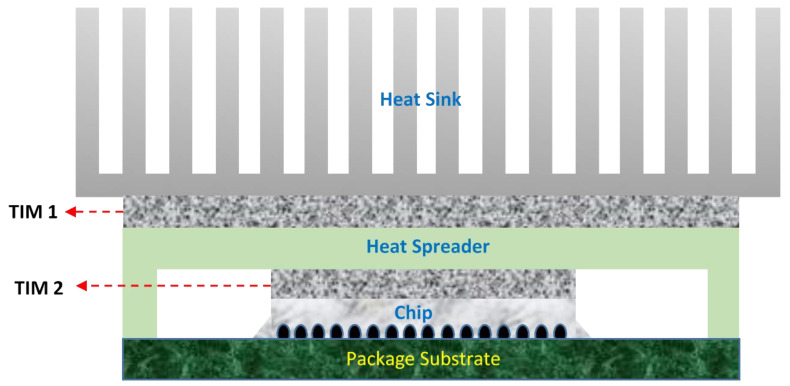
A typical structure of thermal interface materials (TIMs) used in microelectronic packaging.

Thermal interface material (TIM) is an essential part for the efficient extraction of heat generated in semiconductor chips of electronic devices, which are subjected to the thermal cyclic process. The heat transfer across interfaces between materials is becoming a bottleneck for heat conduction considering the increasing trend of miniaturization of microelectronics [1]. In a typical electronics package as illustrated in Figure 1, TIMs are commonly applied at interfaces between two solid modules generally between heat-generating die and heat spreader as well as between the heat spreader and a heat sink. The thermal performance of a TIM is mostly estimated according to its thermal interface resistance (*R_TIM_*), which is a measure of overall resistance to heat dissipation across the interface. It is related to the temperature drop over the interface according to Fourier’s law. After applying a TIM between the solid modules, the effective thermal resistance (*R_TIM_*) at the interface will have two components, i.e., the bulk resistance of the TIM arising from its finite thermal conductivity, *λ_eff_*, and the contact resistance, *R_c_*, between the TIM and the adjoining modules. *R_TIM_* may be expressed as [2]

(1) $$R_{TIM}=\frac{BLT}{\lambda_{eff}}+R_{c1}+R_{c2}$$

where BLT is the bond-line thickness of the TIM and *R_TIM_*, *R_c_*_1_, and *R_c_*_2_ are measured as area-normalized thermal resistances (K m^2^ W^−1^). An ideal TIM should consist of a material combining low BLT with high thermal conductivity and low thermal contact resistance at the interfaces. The goal of the current paper is to reduce *R_TIM_* by designing polymer composite with enhanced *λ_eff_* together with enhanced structural compliance and dielectric characteristics by selecting suitable fillers and polymer matrices. Moreover, the thermal contact resistance between the TIM and attached modules (*R_c_*_1_ and *R_c_*_2_) is also an important factor, which is significantly affected by the type of polymer matrix and filler.

The thermal properties as a primary concern together with contact mechanics and electrical properties are the major considerations in the design of TIMs depending upon the application. Another requirement for TIMs is the appropriate mechanical behavior in terms of good mechanical compliance and wetting capability, or the ability to fill voids leading to an increase in the contact area. The low shear strength for improved formability is a fundamental requirement, which allows required shape change and interfacial contact with the mating modules. For most electronic applications, TIMs are required to have low dielectric constant, high electrical resistance, and high breakdown strength [3]. The loading of inorganic fillers in polymer composite TIMs leads to a significant breakdown field which depends on the content, shape, size, wettability with matrix, dispersion, and electrical properties of these fillers. The mismatch of dielectric constant (for alternating current) or electrical conductivity (for direct current) between the fillers and polymer composites leads to distortion of such an electric field. Therefore, fillers having similar electrical characteristics as polymer matrix TIMs are essential to reduce the intensity of field distortion.

The conventional TIM is composed of thermally conductive polymer composites, which are made of polymer matrices filled by thermally conductive particles (ceramics, carbon-based materials, or metals) to achieve the required thermal conductivity while holding the typical viscous feature of the polymer matrix. Furthermore, the filler type polymer-composite is commonly used as a TIM owing to its electrical insulation and easy processing routes. However, its use in industry is still limited by the low thermal conductivity of polymer as matrix due to its high formability and packing efficiency. To overcome the problem of low thermal conductivity, high thermal conductivity fillers such as ceramics, metals, and carbon-based materials like CNTs, diamond, and graphite are used in polymer matrices. Moreover, numerous techniques such as surface functionalization, filler alignment, and structural optimization are also investigated to enhance the heat conductance through polymer composite TIMs.

Among the available potential fillers used in polymer-based TIMs, ceramics such as alumina (Al_2_O_3_), silicon nitride (Si_3_N_4_), silicon carbide (SiC), boron nitride (BN), and aluminum nitride (AlN) are widely used owing to their stability, desired electrical insulation, and reasonably high thermal conductivity. Alumina (Al_2_O_3_) is the most used candidate filler due to its low cost and relatively high thermal insulation despite its comparatively low thermal conductivity (~30 W m^−1^ K^−1^) [4]. Many other candidate fillers such as carbon nanotubes/nanofibers [5] and graphite/graphene [6] are also used as fillers in TIMs owing to their extremely high thermal conductivities. Despite the graphene monolayer being reported to have very high thermal conductivity (~1000 W m^−1^ K^−1^) at room temperature, its thermal conductivity degrades tremendously due to intrinsic ambient scattering when assembled in bundles. A significantly varying intrinsic thermal conductivity of graphite due to its anisotropic nature poses another challenge to its use as a TIM. The thermal interface between the matrix and fillers further leads to scattering and thus compromises the benefit of these high thermally conductive fillers. For example, it is reported that graphene–polymer and CNT–polymer composites often have thermal conductivity lower than 1 W m^−1^ K^−1^ despite non-dilute concentrations which are associated with weak van der Waals bonding at the filler interfaces where the phonon transport is largely impeded [3,7]. Huang et al. [8] reported that controlling SiC nanowires orientation in epoxy composites can exhibit ultra-high in-plane thermal conductivity (~10 W m^−1^ K^−1^) at very low filler concentration (5 wt%) as compared to random loading of SiC nanowires in epoxy which resulted in only 1.78 W m^−1^ K^−1^.

Polymer composite TIMs are the most widely used candidates as they are generally soft and flexible to overcome the mechanical issues such as delamination, cracking, and void formation when electronic packages undergo a temperature change leading to thermal stresses, due to mismatch in coefficient of thermal expansion (CTE) of different interfaces [9]. Therefore, maintaining a low elastic modulus of polymer composites when hard fillers are loaded is desired to enable better filling with the voids between the TIM and substrates. Moreover, a reduced CTE is also needed to reduce the mismatch with the adjoining ceramic semiconductor interfaces, which leads to reducing levels of induced thermal stresses. The major limitation in thermal performance as a result of using polymers in TIMs is the extremely low thermal conductivity of (~0.1–0.3 W m^−1^ K^−1^). To compensate for this limitation, significant efforts are made to use the polymer TIMs with the addition of suitable fillers. Some common fillers reported in the literature include silicone [2], polyethylene [10], polypropylene [11], polyamide [12], polyvinyl chloride, and epoxy resins [13]. Ralphs et al. [14] reported that magnetically induced percolation of nickel particles in a silicone matrix doubles the thermal conductivity of the composite owing to increases in contact between particles.

The effective properties of composites are interrelated functions of their constituents at atomic and microstructural levels. For example, the effective thermal conductivity of filler-type TIMs is a function of intrinsic thermal conductivities of matrix and filler, volume of filler, and thermal interface resistance (also called kapitza resistance) between the fillers and matrix. This resistance further depends on the size, shape, and surface conduction of the filler material. Tremendous experimental hit-and-trial efforts and cost are involved in selecting the right combinations of various attributes of these constituents to achieve target properties application. A great deal of research is currently focused on developing computational tools, which can predict tailored effective functional properties of the intended composites.

The effective medium theory was initiated by Maxwell for the estimation of properties in composites with noninteracting spherical (3D) fillers. Various modifications of Maxwell’s model have been reported over time. For instance, Bruggeman’s model [15] was modified to allow the model for non-dilute concentrations of fillers, Hamilton and Crosser’s [16] modified Maxwell’s model to include a generalized shape factor, and Hasselman and Johnson (HJ) [17] include the effect of interfacial thermal resistance (ITR). Later on, Every et al. [18] modified the Bruggeman’s model to include HJ’s interface thermal resistance, which is then modified by Jiajun et al. [19] to include a sphericity-dependent factor with the capability to handle varying filler shapes. Nan et al. [20] published an effective medium theory considering aspect ratio and orientation of the fillers with the assumption of regularly shaped ellipsoid fillers and dilute concentrations. Afterward, their model was modified by Siddiqui et al. [21] and Raza et al. [22] for nonuniformly distributed dilute hybrid fillers and percolating dilute hybrid fillers, respectively. The original Nan’s model and its extensions apply to a wide range of filler particle shapes, but these models are restricted to dilute concentrations of fillers. The model is extended in this work to consider non-dilute filler concentrations (high volume loading) using the differential scheme for predicting the effective thermal conductivity of filler-laden TIMs.

The current paper is focused on a design strategy to develop novel filler-laden polymer TIMs with enhanced thermal conductivity and tailored coefficient of thermal expansion (CTE) and shear modulus (G) as functional properties. The implementation of a computational design framework based on modified differential effective medium and mean-field homogenization is explained. Several combinations of polymer matrices and fillers with dilute to non-dilute concentrations, particle size, and other material attributes are considered to attain the target properties of TIMs. Some representative composite samples are synthesized in line with the predictions and the resulting properties are measured for validation.

## 2. Computational Models

### 2.1. Differential Effective Medium Approximation for Effective Thermal Conductivity

Different extensions of Nan’s Model [20] have been previously reported by the author and his co-workers [22,23]. The original model and its extensions apply to a wide range of filler particle shapes, but these models are limited to dilute concentrations of fillers. The existing model is modified based on Bruggeman’s differential effective medium theory in which non-dilute filler concertation (the higher filler loading), is achieved by considering the differential form of low volume fraction relationship and integrating the effects of small increments. In the differential scheme, the higher volume fractions or the non-dilute concentrations are achieved by integrating the effects of small increments. Therefore, for low volume fractions, the effective thermal conductivity equation for dilute concentration can be simplified for small *φ* as

(2) $$\lambda_{eff,11}=\lambda_{eff,22}=\lambda_{m}\left(1+\sum_{i=2}^{N}\frac{\varphi^{i}}{2}\left[\beta_{11}^{i}\left(1+{\langle \cos^{2}\theta \rangle }^{i}\right)+\beta_{33}^{i}\left(1-{\langle \cos^{2}\theta \rangle }^{i}\right)\right]\right)$$

For Equation (2), using the analogy of Every’s model [18] the differential form and its integral form for a single filler can be written as follows in Equations (3) and (4), respectively.

(3) $$d\lambda =\left(\frac{d\varphi }{1-\varphi }\right)(\frac{\lambda }{2})\left(\beta_{11}^{}\left(1+\langle \cos^{2}\theta \rangle \right)+\beta_{33}^{}\left(1-\langle \cos^{2}\theta \rangle \right)\right)$$

(4) $$\int_{\lambda_{m}}^{\lambda_{eff}}\frac{2}{\lambda \left(\beta_{11}^{}\left(1+\langle \cos^{2}\theta \rangle \right)+\beta_{33}^{}\left(1-\langle \cos^{2}\theta \rangle \right)\right)}d\lambda =\int_{0}^{\varphi }\left(\frac{1}{1-\varphi }\right)d\varphi$$

Equation (4) can be solved to result as

(5) $${\left(\frac{\lambda_{eff,11}}{\lambda_{m}}\right)}^{2}=\frac{1}{{\left(1-\varphi \right)}^{\left(\beta_{11}^{}\left(1+\langle \cos^{2}\theta \rangle \right)+\beta_{33}^{}\left(1-\langle \cos^{2}\theta \rangle \right)\right)}}$$

Equation (5) can be solved iteratively for *λ_eff,_*_11_. On the same lines, Equation (3) can be derived to give Equation (6) that can also be solved iteratively for *k_eff,_*_33_.

(6) $${\left(\frac{\lambda_{eff,33}}{\lambda_{m}}\right)}^{2}=\frac{1}{{\left(1-\varphi \right)}^{\left(\beta_{11}^{}\left(1-\langle \cos^{2}\theta \rangle \right)+\beta_{33}^{}\langle \cos^{2}\theta \rangle \right)}}$$

*λ_eff,kk_* in Equations (1)–(6) represents the effective thermal conductivity of composite along global *i^th^* (*x*, *y*, or *z*) axis. *λ_m_* is the thermal conductivity of the matrix, while *φ* and $\left\langle \cos^{2}\theta \right\rangle$ are the volume fraction and orientation factor of the fillers, respectively. The orientation factor, $\left\langle \cos^{2}\theta \right\rangle$, is defined by Equation (7):

(7) $${\langle \cos^{2}\theta \rangle }^{}=\frac{\int \psi (\theta )\cos^{2}\theta \sin\theta d\theta }{\int \psi (\theta )\sin\theta d\theta }$$

where *θ* is the angle between the global *z*-axis and filler’s local *z*-axis. ψ(θ) is the distribution function, which describes the orientation of ellipsoid shaped particulate fillers. $\beta_{kk}^{i}$ is a non-dimensional enhancement factor along the particle’s local *i^th^* axis. It is a function of some materials and processing parameters described by Equation (8):

(8) $$\beta_{kk}^{i}=\frac{\lambda_{e,kk}^{i}-\lambda_{m}}{\lambda_{m}+L_{kk}^{i}\left(\lambda_{e,kk}^{i}-\lambda_{m}\right)}$$

where $\lambda_{e,kk}^{i}$ is the equivalent thermal conductivity of composite unit cell along the particle’s local *i^th^* (*x*, *y*, or *z*) axis as defined in Equation (9). $\lambda_{e,kk}^{i}$ is a measure of filler’s effectiveness for modifying the resulting TC of the composite.

(9) $$\begin{array}{l} \lambda_{e,11}^{i}=\left\{ \begin{array}{l} \lambda_{p}^{i}/\left(1+\gamma_{11}^{i}L_{33}^{i}\lambda_{p}^{i}/\lambda_{m}\right),\;\;\mathrm{for}\;\mathrm{platelet}\;\mathrm{inclusions}\\ \lambda_{p}^{i}/\left(1+\gamma_{11}^{i}L_{11}^{i}\lambda_{p}^{i}/\lambda_{m}\right),\;\;\mathrm{for}\;\mathrm{other}\;\mathrm{shapes} \end{array}\right.\\ \lambda_{e,33}^{i}=\left\{ \begin{array}{l} \lambda_{p}^{i}/\left(1+\gamma_{33}^{i}L_{11}^{i}\lambda_{p}^{i}/\lambda_{m}\right),\;\;\mathrm{for}\;\mathrm{cylindrical}\;\mathrm{inclusions}\\ \lambda_{p}^{i}/\left(1+\gamma_{33}^{i}L_{33}^{i}\lambda_{p}^{i}/\lambda_{m}\right),\;\;\mathrm{for}\;\mathrm{other}\;\mathrm{shapes} \end{array}\right. \end{array}$$

where *λ_p_* is the thermal conductivity of particulate fillers and *L_kk_* is the depolarization factor along filler’s local *i^th^* axis as defined by Equation (10) [24]. $\gamma_{kk}^{i}$ is a non-dimensional parameter that includes the combined effect of Kaptiza radius and particle’s geometry as defined by Equation (11).

(10) $$L_{11}^{i}=L_{22}^{i}=\left\{ \begin{array}{l} \frac{\xi^{i}{}^{{}^{2}}}{2\left(\xi^{i}{}^{{}^{2}}-1\right)}-\frac{\xi^{i}}{2{\left(\xi^{i}{}^{{}^{2}}-1\right)}^{3/2}}\cosh^{-1}\xi^{i},\;\;\;\;\mathrm{for}\;\xi^{i}\geq 1\\ \frac{\xi^{i}{}^{{}^{2}}}{2\left(\xi^{i}{}^{{}^{2}}-1\right)}+\frac{\xi^{i}}{2{\left(1-\xi^{i}{}^{{}^{2}}\right)}^{3/2}}\cos^{-1}\xi^{i},\;\;\;\;\mathrm{for}\;\xi^{i}<1 \end{array}\right.$$

(11) $$\gamma_{kk}^{i}=\left\{ \begin{array}{l} \left(2+1/\xi^{i}\right)\alpha_{k},\;\;\mathrm{for}\;\xi^{i}\geq 1\\ \left(1+2\xi^{i}\right)\alpha_{k},\;\;\mathrm{for}\;\xi^{i}<1 \end{array}\right.$$

With $\xi^{i}$ being the aspect ratio of ellipsoid shaped particulate fillers taken as *r*_3_*/r*_1_, where *r*_3_ and *r*_1_ are the radii along filler’s *z* and *x* directions, respectively. Besides, *α_k_ = (λ_m_R_int_*)/*a_k_* is Kapitza radius with *R_int_* is taken as thermal interface resistance and *a_k_* is the radius of *_i_*_th_ ellipsoid shaped filler along the *k_th_* axis. Note that *r*_3_ represents the thickness for oblate particles (such as platelets or discs) or length for prolate particles (such as fibers).

### 2.2. Mean-Field Homogenization Scheme for Elastic Modulus and Coefficient of Thermal Expansion

Akhtar et al. [23] have found a good agreement between the experimentally measured CTE and the one predicted by mean-field homogenization of Mori-Tanaka. According to the scheme, the effective elasticity tensor and CTE are estimated using the Equations (12)–(14):

(12) $$C_{eff}=p_{i}^{}C_{i}:\;A_{i}+(1-p_{i}^{})C_{m}:A_{m}$$

(13) $$\alpha_{eff}=\alpha_{i}I_{2}+p_{i}^{}(C_{i}^{-1}-C_{m}^{-1})W{\left((1-p_{i}^{})I_{4}+p_{i}^{}W\right)}^{-1}$$

(14) $$\begin{array}{l} A_{m}={\left[(1-p_{i}^{})I_{4}+p_{i}^{}B_{a}\right]}^{-1}\\ A_{i}=B_{a}:\;A_{m}\\ B_{a}=[I_{4}+S:C_{m}^{-1}(C_{i}-C_{m})]-1\\ W=C_{i}A_{i}C_{m}^{-1} \end{array}$$

*C_eff_* and *α_eff_* are the homogenized stiffness tensor and the effective CTE of the resulting composite, respectively. *C_i_* and *C_m_* are the stiffness tensors of inclusion and matrix, respectively. *A_m_* and *A_i_* are the strain localization tensors; *I*_2_ and *I*_4_ are the 2nd order and 4th order identities, respectively; and *S* is the Eshelby’s tensor.

### 2.3. Selection of Potential Fillers and Polymers

Polymers are used as a matrix in composites as TIM due to their electrical insulation, formability, and filling efficiency when applied as an interface between modules. To increase the thermal conductivity of matrix material, thermally conductive fillers are essential. Moreover, one must choose fillers having similar electrical characteristics as polymers to maintain the high breakdown strength, which is the intrinsic property of polymers and one of the major reasons for their use as TIMs. Numerous polymers are considered in the current work as potential candidates based on their use in the TIM industry. These include polypropylene (PP), high-density polyethylene (HDPE), thermoplastic polyurethane (TPU), polysulfone (PSU), epoxy, and silicone. The properties of these polymers are shown in Table 1 [25,26,27,28]. To fulfill the requirements of the TIMs, several conventional and emerging ceramic and carbon-based fillers are considered as potential candidates considering their thermal conductivity, electrical insulation, stability, and dielectric properties. Due to the high electrical conductivity, metallic fillers are not considered in the current design. The candidate potential fillers and their properties are listed in Table 1. Aluminum nitride (AlN), silicon nitride (Si_3_N_4_), boron nitride (BN), and silicon carbide (SiC) are included as fillers due to their electrical insulation, stability, and reasonably high thermally conductivity. Despite its relatively low thermal conductivity, alumina (Al_2_O_3_) is considered owing to its low cost and high electrical resistivity. A semiconductor gallium nitride (GaN) is considered as emerging new high thermal conductivity material to see its usability as filler material.

## 3. Materials and Experimental Methods

### 3.1. Materials

Several polymer composite systems with different matrices and filler attributes are synthesized to validate the proposed computational design. The composite development included pretreatment of filler’s particles, melt mixing of the ingredients, compression molding, measurements, and microscopy. Al_2_O_3_ and AlN are selected as fillers, while HDPE, PP, and TPU are found to be the best potential matrix based on composite design. AlN powders and HDPE granules are procured from Surmet Corporation (Burlington, MA, USA) and SABIC (Riyadh, KSA) respectively, while Al_2_O_3_ powders, γ-methylacryloxypropyl trimethoxy silane (for surface treatment), and PP granules are purchased from Sigma Aldrich (St. Louis, MO, USA). PU was supplied by Taiwan PU Corporation (New Taipei City, Taiwan). The filler particles were surface treated according to the method reported earlier [29] to improve interface compatibility with the polymer matrix. A 2 wt% γ-methylacryloxypropyl trimethoxy silane was added to ethanol and mixing was performed for 3 min at room temperature. A gradual dropwise addition of HCL was then performed to adjust the pH to a value of 5 followed by stirring for 10 min. The preweighed ceramic powders (Al_2_O_3_ or AlN) were then added to the prepared solution and stirred for 20 min at room temperature followed by additional stirring for 40 min at 70 °C. The treated particles were then allowed to settle down for 1 h before pouring out. Overnight drying of particles was then performed at 90 °C using vacuum oven. The treated powders were then stored in vacuum desiccator to avoid dust or moisture contamination.

### 3.2. Development of Composites

The preweighed amounts of polymer granules and pretreated ceramic powders (Al_2_O_3_ or AlN) were melt-mixed as per designed volume fractions at 150 °C with 60 rpm for 20 min using Brabender Measuring Mixer 50 EHT. After attaining the set temperatures (range: 150–180 °C) in the mixing chamber, the polymer granules and preweighed ceramic powders were added at a slower rpm (30 rpm) followed by mixing the two phases at 60 rpm for 30 min. These composite lumps were then processed further to make samples of the desired shapes for measurements. Preweighed lumps are then hot-pressed in a compression molding machine at the melting temperature of the matrix for the particular composition in the form of disc-shaped samples (31 mm diameter) with the required thickness.

### 3.3. Testing

The thermal conductivity of samples is measured using TCi Thermal Conductivity Analyzer by C-Therm Technologies Ltd., Fredericton, NB, Canada. This equipment measures the thermal conductivity based on a modified transient plane source method where a one-sided interfacial heat reflectance sensor is employed with a transitory constant heat source to the specimen. The thermal expansion coefficient of composites is measured using a METTLER TOLEDO Thermal mechanical analyzer (TMA/SDTA 1 LF/1100, METTLER TOLEDO, Columbus, OH, USA). This equipment measures the dimensional changes in the composite as a function of temperature. The resulting displacement as a result of dimensional changes is detected by the probe placed on the sample by means of linear variable differential transformer (LVDT) sensor connected to the other end of the probe. A very small sample with dimensions 3 mm × 3 mm × 2 mm was cut to meet the size requirement of the equipment. Filler’s morphology and their distribution and interface condition in the composites were observed by Tescan Lyra 3 Field Emission Scanning Electron Microscope (FESEM, Tescan, Brno, Czech Republic). The powders and fractured surfaces for microscopy are gold-coated using a sputter coater for 20 s, and FESEM was operated in BSE mode at 20 kV for all composite samples.

## 4. Results and Discussion

Computational results are first presented to analyze and predict properties with different combinations of potential polymer and fillers to develop new TIMs. The aim is to elucidate the effect of intrinsic properties and attributes of individual phases in the resulting composites. Characterization and property measurement techniques are then used to validate the predictions under varied conditions. The results are presented here to design promising benchmark TIMs. To design the best combinations of fillers and matrices, the target values of properties are established based on the requirements of emerging TIMs. Thermal conductivity in the range of 1 to 2 W m^−1^ K^−1^ is considered a typical desired value [3] for polymer-based TIMs, and thus a value of 1.5 W m^−1^ K^−1^ is set as a threshold value. The threshold values to obtain the desired structural properties for the TIMs are determined based on material properties acquired from literature. A maximum value of shear modulus in the range of 0.5 to 0.6 GPa and a minimum value of CTE less than 100 K^−1^ are considered as target values in the computational design to achieve the filler–matrix combination to satisfy the structural properties of intended TIMs.

### 4.1. Prediction of Minimum Filler Size

The author and his co-workers have previously reported [22,29] that the particle size and thermal interface resistance between the matrix and filler are the dominant and most sensitive parameters in controlling the effective thermal conductivity of a particular single and hybrid filler polymer composite. These two parameters play a major role in controlling the effective thermal conductivity of the composite through a non-dimensional parameter *α_k_* as defined in Equation (11), also called the effect of Kaptiza radius, where the particle’s radius (*a_k_*) is the decision-making parameter. The maximum allowed ratio of interface thermal resistance and particle radius (*R_int_*/*a_k_*), which leads to finding minimum filler size, is determined. This depends on the intrinsic thermal conductivity of the polymer matrix and filler. After identifying the minimum particle size, the effect of filler loading is then studied to achieve the target properties of the intended composites.

**Figure 2:**
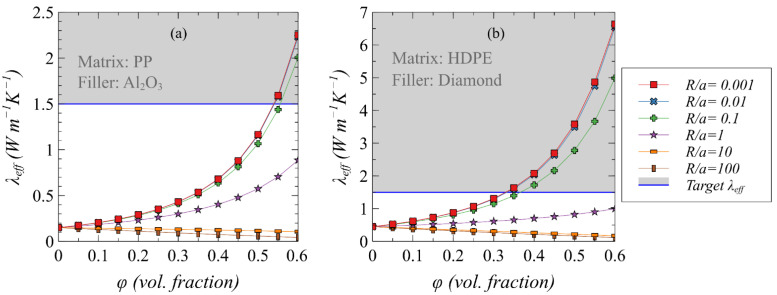
The predicted effective thermal conductivity considering the lowest and highest intrinsic thermal conductivity combinations of matrices and fillers with different *R_int_*/*a_k_* ratios. (**a**) PP with spherical Al_2_O_3_ fillers. (**b**) HDPE with spherical diamond particulates.

Two different combinations of matrices and fillers with the lowest and highest intrinsic thermal conductivity values are used to predict the target effective thermal conductivity of composites as a function of volume loading at different *R_int_*/*a_k_* ratios. Figure 2 shows the variation of thermal conductivity for these two combinations. A composite system with PP as matrix and spherical Al_2_O_3_ as filler is used keeping in view their lowest intrinsic thermal conductivity (Figure 2a) while HDPE laden with spherical diamond is considered as a second combination due to the highest thermal conductivity values (Figure 2b) to capture the threshold particle size. As shown, the onset of enhancement in effective thermal conductivity is predicted at a maximum ratio of unity in both cases, i.e., *R_int_*/*a_k_* = 1. A value of *R_int_*/*a_k_* = 0.1 is predicted to bring a significant increase in the effective thermal conductivity as compared to the pristine polymer matrix.

The value of *R_int_* for a particular composite system depends upon the inherent properties of the matrix such as specific heat, density, and Debye velocity, and interface dependent properties such as acoustic impedance and critical angle for phonon–phonon coupling. For the polymers, having smaller bulk densities and large heat capacities, the value of *R_int_* typically is determined from the ratio of phonon velocities of the matrix and fillers. Based on the phonon velocities of ceramics (such as AlN and Al_2_O_3_) and polymers (such as HDPE and PP), the maximum variation of *R_int_* for the ceramic–polymer composite systems is expected to be within a range of 1.15 × 10^−7^ to 22.5 × 10^−7^ m^2^ K W^−1^ [30]. The reported values of *R_int_* are mostly based on the calibration of theoretical models with empirical data, as the exact phonon velocities are seldom available. A value of 3.32 × 10^−7^ m^2^ K W^−1^ is assumed in this current work, which is reported for a similar composite system, keeping in view the surface treatment of fillers in the current experimental work. This results in a minimum required particle’s radius of 3 µm based on *R_int_*/*a_k_* = 0.1.

### 4.2. Effect of Polymer–Filler Combinations on Effective Thermal Conductivity

**Figure 3:**
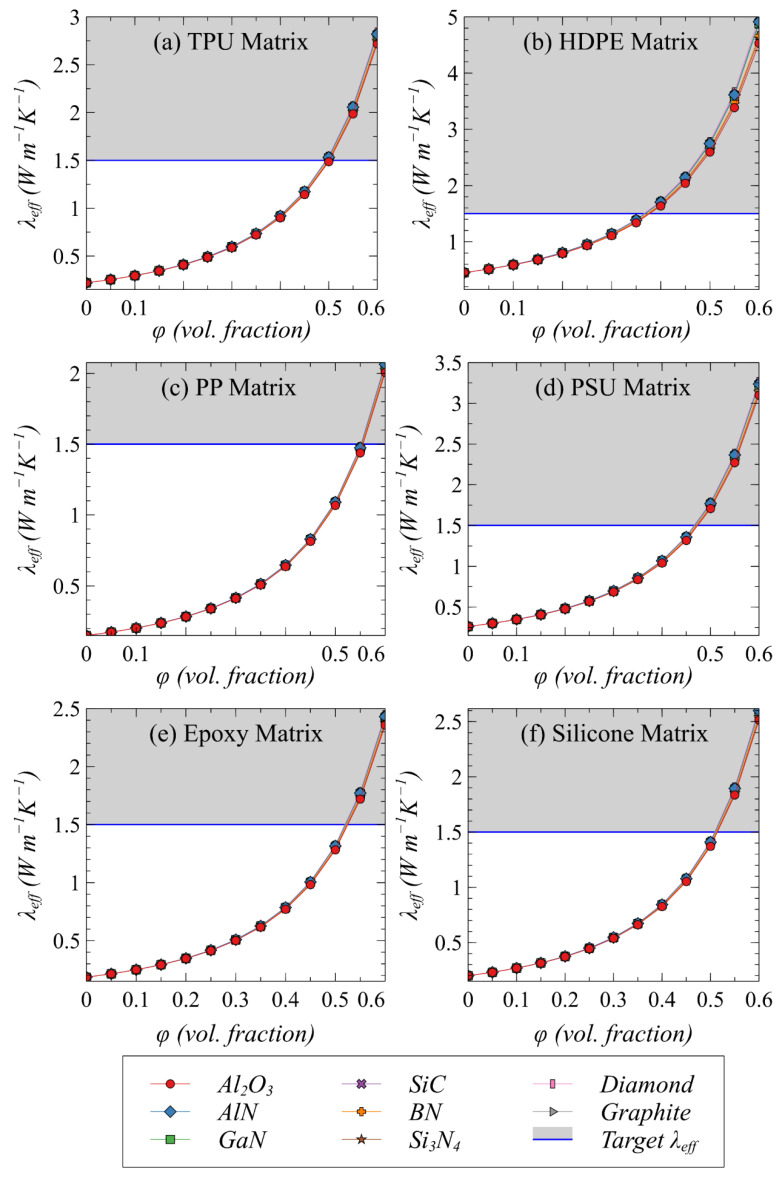
The predicted effective thermal conductivity of composites with different polymer–filler combinations at *R_int_*/*a_k_* = 0.1.

The effect of potential fillers loaded in candidate polymers (as depicted in Table 1) on the effective thermal conductivity of resulting composite systems is studied based on the identified minimum particle size. Figure 3a–f shows the predicted thermal conductivity variation as a function of the volume fraction of selected fillers in six types of polymer matrices as potential TIMs. Note that increasing the loading of fillers in any particular polymer matrix tends to increase effective thermal conductivity irrespective of the filler type despite a wide range of their intrinsic thermal conductivity values. The effective thermal conductivity is predominantly governed by the polymer (conducting phase) and any ceramic and carbon-based filler can be used. This is associated with a very large difference in the thermal conductivity values of the continuous (polymer) and discontinuous (filler) phase.

It is evident from Figure 3 that HDPE is the most suitable polymer among the available candidates, where a minimum filler loading of 35 vol% is sufficient to achieve the target thermal conductivity of 1.5 W m^−1^ K^−1^. An ultra-high thermal conductivity of 5 W m^−1^ K^−1^ is possible in an HDPE-based TIMs if the loading of fillers is increased to 60 vol%. The next best polymer candidates are PSU and TPU, wherein a thermal conductivity of ~3 W m^−1^ K^−1^ is predicted at a filler loading of ~60 vol%. The target threshold value of 1.5 W m^−1^ K^−1^ is possible with a filler loading of ~45 vol% in these two polymers. It is also evident that conventionally used epoxy- and silicone-based composites as TIMs are limited to a maximum value of ~2 W m^−1^ K^−1^ even with a very high loading of ~50 vol%, owing to their low intrinsic thermal conductivity. A very high concentration of fillers is needed in PP to achieve the threshold effective thermal conductivity until a larger particle size and/or high aspect ratio is used, which is confirmed in the validation section. Note that filler attributes such as larger particle size, high aspect ratio, and/or hybrid fillers can be used to decrease the required volume fraction to achieve any particular thermal conductivity [29,31] and thus further design optimization is possible. Apart from the thermal response, note that other requirements such as mechanical compliance and dielectric characteristics of TIMs should not be compromised merely based on achieving high thermal conductivity. Therefore, a further investigation is essential to compare polymer–filler combinations based on mechanical properties, which is discussed next.

### 4.3. Effect of Polymer–Filler Combinations on Effective Shear Modulus and Coefficient of Thermal Expansion

**Figure 4:**
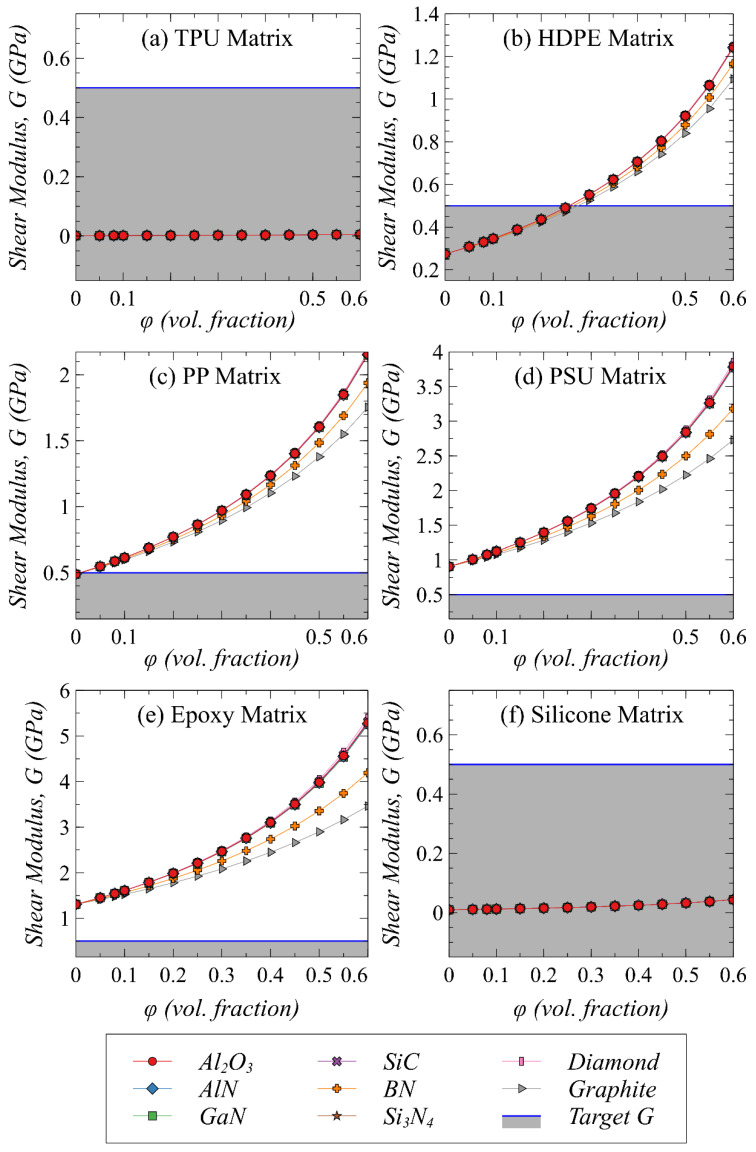
The predicted effective shear modulus of composites with different polymer–filler combinations.

**Figure 5:**
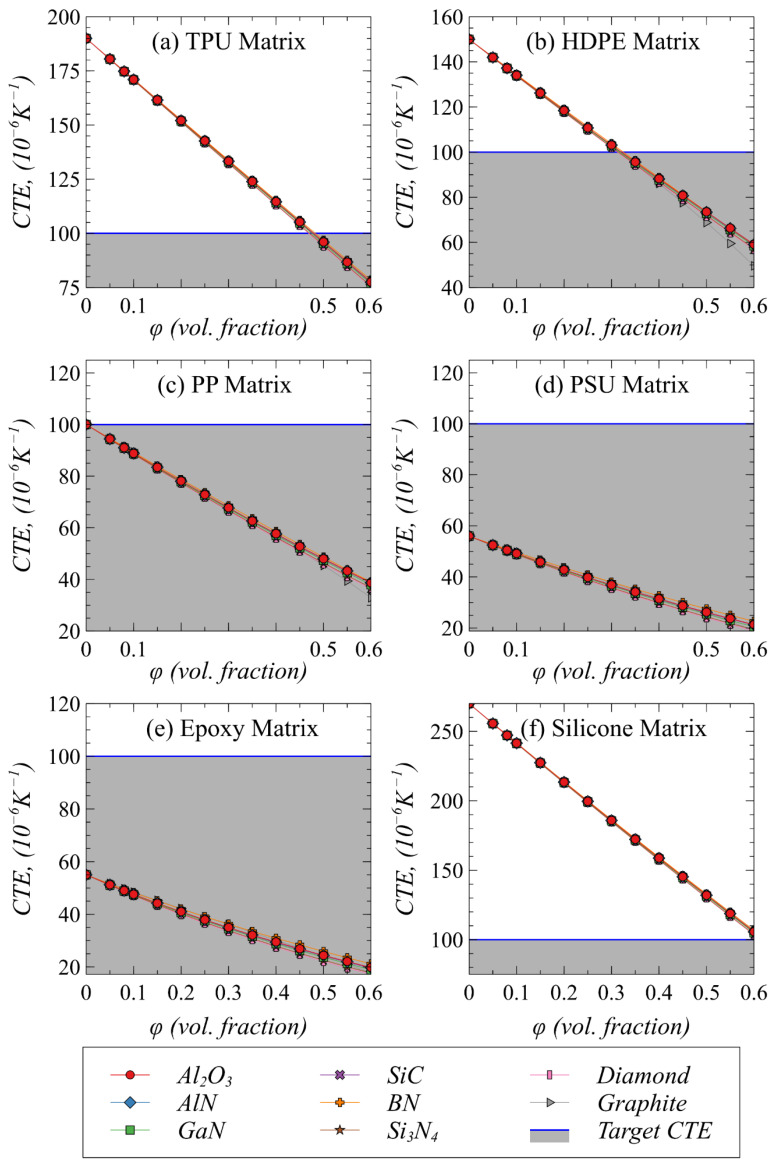
The predicted effective coefficient of thermal expansion of composites with different polymer–filler combinations.

The TIMs made of polymer composites should demonstrate good mechanical compliance leading to fill out the voids when applied in between low-CTE modules in electronic packages. This leads to the most fundamental engineering requirements, i.e., (1) low shear modulus to allow shape change and improved adhesion and (2) lower CTE to minimize the mismatch in CTE with the chips and heat spreaders in the package to avoid thermos-mechanical stresses. Therefore, the effect of selected fillers loaded in potential polymers on effective G and CTE are studied as a function of volume fraction. Figure 4 and Figure 5 show the effective G and CTE as a function of volume fraction of selected fillers in potential polymer matrices, respectively. It can be observed from Figure 4 that TPU and silicone maintain their low G values despite adding very high loading of any kind of filler, which is associated with very low intrinsic G value of these matrices. However, their relatively higher CTE especially in the case of silicone tends to be a restricting factor as shown in Figure 5. A very high loading (>50 vol%) of filler in silicone would be needed to satisfy the requirement of reaching a set CTE value of <100 × 10^−6^ K^−1^. On the contrary, the G value tends to increase as a function of filler volume fraction when epoxy, PSU, and PP are used as matrix. Therefore, the intended TIM composite would not be able to maintain the required maximum value of 0.5–0.6 GPa in such conditions. This increasing trend in G is relatively less in the case of BN and graphite fillers as compared to the other fillers, which is associated with their small intrinsic G values. Nevertheless, the relatively low inherent CTE values particularly of PSU and epoxy are some of the attractive choices to be used as TIMs. As shown in Figure 5, the effective CTE of composites in the case of epoxy and PSU tends to decrease which are expected to reduce the thermal stresses because of less mismatch with the adjoining modules. Among all the potential polymer candidates, HDPE is predicted to be the best candidate for attaining desired CTE and G when loaded with fillers with a minimum of 30 vol% and a maximum of 45 vol%. Therefore, there is a trade-off between achieving ultra-high thermal conductivity and maintaining a low modulus value when filler concertation exceeds 40% in HDPE.

The forgoing investigation leads to some important findings, which are considered important for TIM designers. The effective thermal and structural properties mainly depend on the continuous polymer matrix phase, while most of the fillers have a similar effect on the effective properties. The CTE of the listed polymers is significantly higher as compared with the potential fillers while the opposite is true in the case of G. Therefore, when the TIMs are subjected to mechanical and thermal strains, these fillers do not tend to deform and most of the deformation is localized to the polymer matrix. In such situations, as reported earlier [29], the major strengthening mechanism is dominated by limiting the motion of polymer molecular chains at the interfacial boundary with the fillers which require reasonably good interfacial strength. As far as filler selection is concerned, a decision can be further made based on other criteria such as cost, availability, and most importantly the similar electrical properties to the polymer matrix such that to achieve high breakdown strength in the resulting composites.

### 4.4. Microscopic Analysis of Fillers and Synthesized Composites

**Figure 6:**
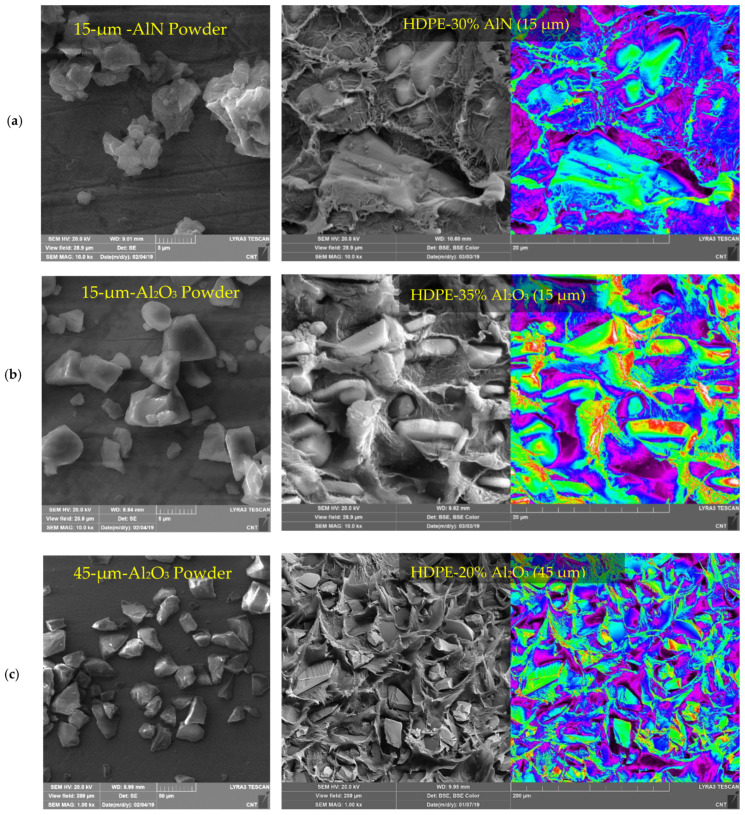
Characterization of ceramic fillers and fractured HDPE-based composites. FESEM images of HDPE/ceramic composite at (**a**) 30 vol% AlN (15 µm) loading, (**b**) 35 vol% Al_2_O_3_ (15 µm) loading, and (**c**) 20 vol% Al_2_O_3_ (45 µm) loading.

**Figure 7:**
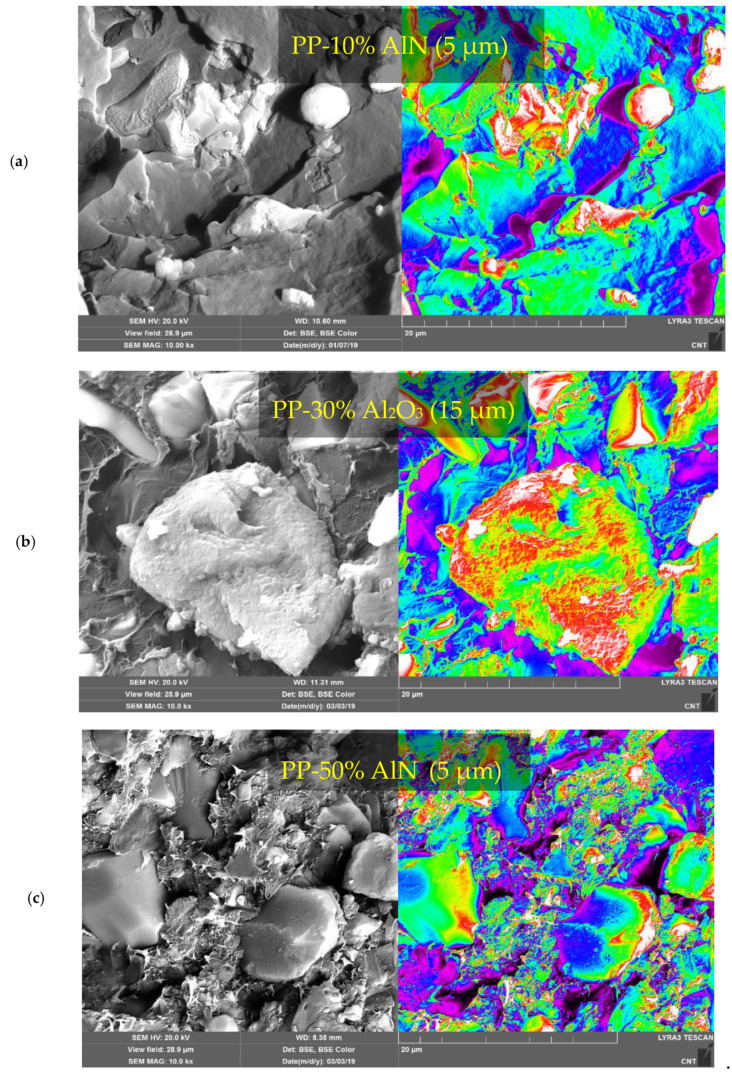
Characterization of fractured PP-based composites. FESEM images of PP/ceramic composite at (**a**) 10 vol% AlN (5 µm) loading, (**b**) 30 vol% Al_2_O_3_ (15 µm) loading, and (**c**) 50 vol% AlN (5 µm) loading.

To validate the model predictions, filler-filled polymer composites in low- and high-volume fraction are developed using different matrices and filler attributes in line with predictions. Table 2 shows the details of these samples. Figure 6 shows some representative FESEM images of HDPE composites with Al_2_O_3_ and AlN as fillers in different loadings and sizes, while Figure 7 shows images of PP loaded with Al_2_O_3_ and AlN. The images of A_2_O_3_ and AlN powders as shown in Figure 6 conform to the reported sizes and shapes. The bigger 45 µm Al_2_O_3_ are roughly equiaxed, while 15 µm Al_2_O_3_ particles are mostly oblate with a large aspect ratio. A backscattered electron (BSE) mode is used to capture the composites images for achieving better contrast of fillers in the matrices. In general, no porosity is observed, and particles are distributed homogeneously in the matrices, which shows the effectiveness of the processing route used in this work. The BSE color images enable the ceramic particulates to be identified within the matrix. Owing to the higher average atomic weight of ceramic particles, they appear brighter as compared to the polymer matrix. The contour images of these images further clarify the fillers’ existence in the matrix, which appears as greenish-red in the bluish matrix due to higher signal density.

### 4.5. Validation of Computation Design

**Figure 8:**
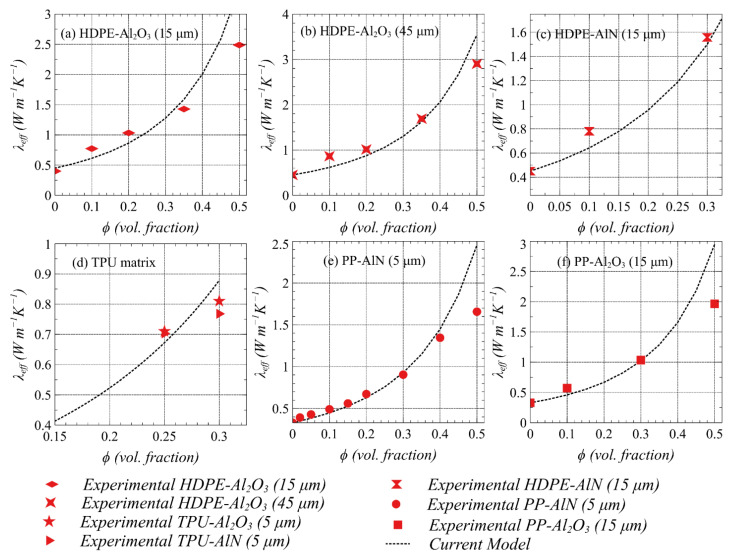
The measured and predicted thermal conductivity of different type filler and matrix composites as a function of filler loading. (**a**) HDPE/Al_2_O_3_ composites with 15 µm Al_2_O_3_. (**b**) HDPE/Al_2_O_3_ composites with 45 µm Al_2_O_3_. (**c**) HDPE/AlN composites with 15 µm AlN. (**d**) TPU with AlN and Al_2_O_3_ composites with 5 µm filler. (**e**) PP/AlN composites with 5 µm AlN. (**f**) PP/Al_2_O_3_ composites with 15 µm Al_2_O_3_.

Figure 8a–f shows the measured and predicted thermal conductivity as a function of fillers loading from low to very high concentration in different composite systems with different attributes. These composite systems include several combinations of HDPE, TPU, and PP as a matrix with Al_2_O_3_ and AlN as fillers with varying particle sizes as described in Table 2. The comparison of measured and predicted thermal conductivity, in general, confirms that the proposed differential effective medium approach is accurate enough in predicting the thermal response of the polymer composites for intended TIM. This is associated with the fact that the interactions amongst effective fields of the neighboring particles are nicely captured by the current model owing to the integration of the infinitesimally dilute additions of fillers into the homogeneous matrices. The results therefore indicate that the proposed non-dilute model has overcome the deficiencies typical to dilute effective medium theories, which underestimate the thermal conductivity, particularly at higher filler loading.

As shown in Figure 8a–c, HDPE-based composites loaded with Al_2_O_3_ (sizes: 15 µm and 45 µm) and AlN (size: 15 µm) exhibit higher thermal conductivity for any particular filler volume fraction, and the conductivity increases when the content of the filler increases. On the contrary, the TPU-based composites with 5 µm Al_2_O_3_ and AlN each (Figure 8d) and PP-based composites loaded 5 µm and 15 µm Al_2_O_3_ (Figure 8e,f), result in relatively lower thermal conductivity when compared to HDPE-based composites owing to the smaller inherent thermal conductivity of TPU and PP (continuous phase) as discussed earlier. The effect of filler size on resulting thermal conductivity can also be realized from Figure 8 if we compare small and large particle sizes in HDPE-Al_2_O_3_ composite systems with two different sizes. For example, HDPE/45 µm-Al_2_O_3_ composites can provide higher thermal conductivity as compared to HDPE/15 µm-Al_2_O_3_ composites for any particular filler loading. Note that from Figure 8d those TPU-based composites loaded with 5 µm Al_2_O_3_ and AlN result in almost similar conductivity which confirms that the intrinsic thermal conductivity of continuous polymer matrix has a dominant role in controlling the effective thermal conductivity. Another important factor is found to be the aspect ratio of the particle in controlling the effective thermal conductivity when the PP/5 µm-AlN (equiaxed/near-spherical) composite system is compared with PP/15 µm-Al_2_O_3_ (oblate-shaped) as shown in Figure 8e,f, respectively. It can be inferred from the results that the relative enhancement in effective thermal conductivity in the case of the oblate-shaped particle is more as compared to the spherical one. This is associated with the fact that the oblate (or prolate) particles have more tendency to form an interconnecting network and therefore can provide a higher effective thermal conductivity, which is demonstrated using Equation (9).

As the forgoing results imply, the filler size and aspect ratio directly influence the overall thermal performance of the TIM. Therefore, these filler attributes should be selected carefully such that the mechanical performance of the TIMs should not be compromised. A large filler can result in poor adhesion with the attached electronic modules allowing the formation of voids. On the other hand, very small fillers such as nanoparticles can result in a significant increase in Kapitza resistance due to larger surface area and increased boundary scattering, which lead to impeding heat transfer and thus reduced thermal conductivity of TIM as demonstrated from measurements and predictions. It is also established that the effect of Kapitza resistance can be reduced if filler with a high aspect ratio is used for the same percentage loading.

**Figure 9:**
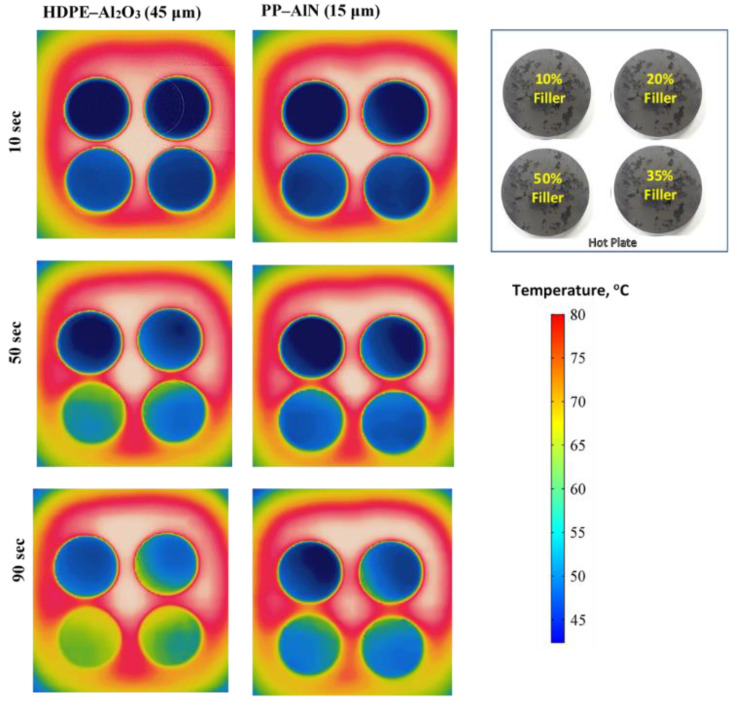
Thermal images of different composites captured as a function of time. HDPE/Al_2_O_3_ composites (45 µm Al_2_O_3_) with 10, 20, 35, and 50 vol% loading. PP/AlN composites (15 µm AlN) with 10, 20, 35 and 50 vol% loading.

To confirm the capacity of thermal conductance through prepared composites thermal imaging is conducted using an infrared camera. Thermal images of HDPE/Al_2_O_3_ (45 µm Al_2_O_3_) PP/AlN composites (15 µm AlN) with 10, 20, 35, and 50 vol% loading captured as a function of time is shown in Figure 9. In general, the composites with higher loading tend to increase transfer heat at a faster rate temperature at a faster rate. The HDPE composite loaded with 45 µm Al_2_O_3_ shows a significantly greater temperature increase as compared to PP composite with 15 µm AlN at any particular loading and time. This qualitative analysis is in agreement with the measured and predicted thermal conductivity of these composite systems and confirms that the polymer matrix as a continuous phase with larger particle size is the dominant factor in adjusting the effective thermal conductivity.

**Figure 10:**
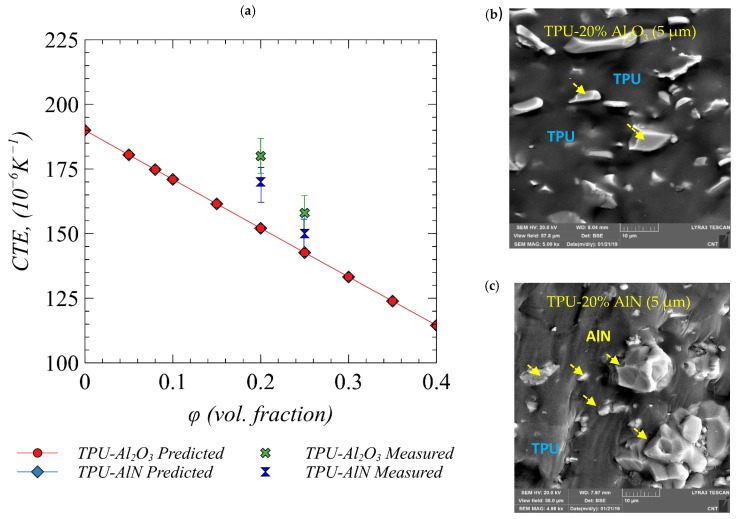
(**a**) The prediction of effective CTE of TPU/Al_2_O_3_ and TPU/AlN composites as a function of filler loading. The measured CTE values of these composites systems with 20 vol% and 25 vol% loadings are also shown. (**b**) FESEM images of TPU/Al_2_O_3_ 20 vol% AlN (5 µm) loading. (**c**) FESEM images of TPU/AlN 20 vol% AlN (5 µm) loading.

A comparison of measured and predicted CTE of AlN- and Al_2_O_3_-loaded TPU composites as a function of volume fraction is shown in Figure 10a. The corresponding FESME images of TPU with 20 vol% Al_2_O_3_ and AlN fillers are also shown in Figure 10b,c, respectively, confirming that there were no defects found in the prepared composites. It can be observed that the ceramic particles have a slightly high aspect ratio and are almost homogeneously dispersed in the polymer matrix. The predictions of CTE are in close agreement with experimentally measured values, and it can be observed that the CTE of the composite can be tailored by suitably loading of low-CTE ceramic fillers. The addition of the ceramic fillers in the polymer matrix tends to decrease the effective CTE as the volume fraction is increased and thereby has the potential to reduce mismatch with adjoining modules in electronic packages, which is one of the requirements of high-performance TIMs. Note that the slight difference between predicted and the measured value is associated with the fact that the model used in the current work is based on a mean-field approach, which assumes spherical fillers randomly dispersed in the matrix.

### 4.6. Effect of Filler–Matrix Interface and Filler Orientation

As demonstrated, higher thermal conductivity fillers lead to the required enhanced thermal conductivity of the TIM but have some drawbacks in terms of mechanical properties predominantly at higher loading. Therefore, attaining the same thermal performance at lower filler loading is desired for designing emerging TIMs. Methods such as enhancing thermal compatibility at the filler–matrix interface and filler alignment are explored. Apart from the filler–matrix combination, filler size, and dispersion, the current computational framework can handle filler orientation and filler–matrix interface effect on the resulting effective thermal conductivity. Some representative published work is considered to validate the model and provide some guidelines for emerging polymer-based TIMs.

**Figure 11:**
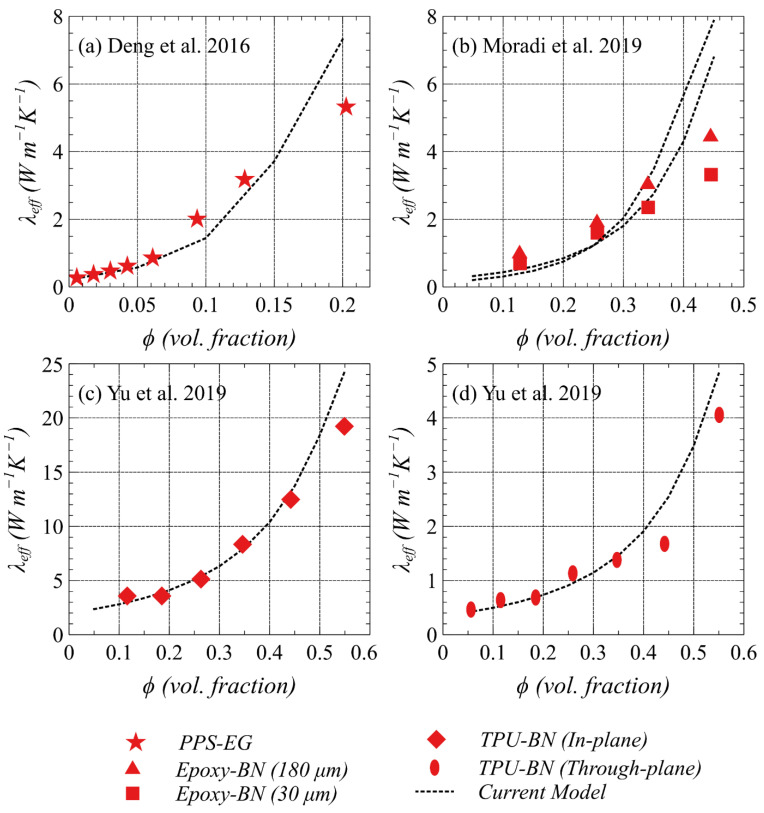
Validation of the current model using experimentally measured thermal conductivity results of different cases of interface thermal resistance and orientation of the fillers.

The crux of the current model is to minimize the percolation threshold for achieving maximum thermal conductivity by tailoring the factors such as particle size, aspect ratio, and preferred filler orientation in addition to the intrinsic properties of fillers and matrices. To this end, the model is validated by experimental data of thermal conductivity with different cases of filler–matrix interface resistance (*R_int_*), and filler orientation ($\left\langle \cos^{2}\theta \right\rangle$) available in the literature [32,33,34]. A comparison of reported experimental results with the current model is presented in Figure 11 to demonstrate the model capability of considering these factors on the resulting effective thermal conductivity for various filler–polymer combinations. The related inputs used are given in Table 3. Deng et al. [34] explored composites with polyphenylene sulfide (PPS) as a matrix and expandable graphite (EG) as a filler, which demonstrated to result in very low *R_int_* due to the strong interaction of PPS and EG as shown in Figure 11a. Moradi et al. [32], on the other hand, showed a high thermal resistance between hexagonal boron nitride (hBN) fillers when loaded in the epoxy matrix as depicted in Figure 11b. Nevertheless, a bigger particle size (180 µm) as compared with a smaller filler size (30 µm) of hBN resulted in higher effective thermal conductivity, which confirms that the interface resistance can be compensated by using a bigger particle size.

Aligning fillers in a polymer matrix for forming continuous heat-conducting paths has recently sought by many researchers, which could lead to a breakthrough in the design of efficient and reliable TIMs. Figure 11c,d shows validation of the current framework with the experimental work of Yu et al. [33] who demonstrated a process-induced in-plane and through-plane alignment of hBN particles in the TPU matrix. The measured thermal conductivity of the composites in the two different orientations is in very close agreement with the current model predictions, which confirms the capability of the current model to include filler orientation in addition to other attributes. It is demonstrated that the fillers with parallel alignments can lead to tremendously high thermal conductivity as compared to unaligned fillers.

## 5. Conclusions

An integrated computational and experimental approach is used to design and develop filler–polymer TIMs by reducing their bulk thermal resistance without compromising the required mechanical response. The modified differential effective medium framework formulated for considering non-dilute filler concentration (>50 vol%) exhibited excellent agreement with experimental data. The mean-field homogenization scheme is used for predicting the structural response of the composites. The effective thermal conductivity is found to be tunable in a wide range by selecting various polymer–filler combinations that maintain the required shear modulus and coefficient of thermal expansion of TIMs. The inherent properties of the pristine polymer represent a controlling factor for modifying the overall thermo-mechanical performance of TIMs. HDPE and TPU filled with randomly dispersed dielectric ceramic fillers with a minimum average particle diameter of 6 µm are the potential combinations exhibiting a thermal conductivity above 4.0 W m K^−1^ with a maximum filler loading of 60 vol%. Various composite systems composed of HDPE, TPU, and PP as polymer matrix-loaded Al_2_O_3_ and AlN fillers with designed attributes are developed for validation. The predictions are in close agreement with measured properties, which confirms the capability of the proposed computational scheme in selecting appropriate polymer and filler combinations for TIMs. The model demonstrated with high accuracy that high aspect ratio fillers, such as oblate-shaped, and in-plane alignment of fillers drastically enhance the effective thermal conductivity even with dilute concentration owing to the formation of the percolating network. The presented integrated material design, development, and testing approach for developing polymer–composite TIMs with tailored thermal and structural properties is novel and has a great potential to benefit the electronic industry and the researchers involved in designing and developing new TIMs.

## Figures and Tables

**Table 1:** Properties of potential matrices and fillers for thermal interface material [25,26,27,28].

Potential Polymers and Fillers Considered	Thermal Conductivity (*W* m^−1^ K^−1^)	Poisson’s Ratio	Elastic Modulus (GPa)	Shear Modulus (GPa)	Coefficient of Thermal Expansion (10^−6^ K^−1^)
**Polymer Matrices**					
**Thermoplastic polyurethane (TPU)**	0.22	0.49	0.0035	0.00112	190
**High-density polyethylene (HDPE)**	0.45	0.46	0.8	0.274	150
**Polypropylene (PP)**	0.15	0.43	1.4	0.49	100
**Polysulfone**	0.26	0.37	2.48	0.905	56
**Epoxy**	0.185	0.32	3.45	1.31	55
**Silicone**	0.2	0.47	0.028	0.0094	270
**Fillers**					
**Alumina (Al_2_O_3_)**	33	0.21	370	88.0	4.6
**Aluminum Nitride (AlN)**	177	0.23	330	126	4.3
**Gallium Nitride (GaN)**	130	0.25	306	122.4	3.1
**Silicon Carbide (SiC)**	170	0.21	430	177.7	4.0
**Boron Nitride (BN)**	52	0.21	41	41.0	6.0
**Silicon Nitride (Si_3_N_4_)**	43	0.23	310	65.3	1.4
**Diamond**	2000	0.2	850	440	0.8
**Graphite**	180	0.2	21	8.75	4.9

**Table 2:** Description of prepared polymer composite samples.

Matrix	Fillers	Mean Particle Size (µm)	Composition, *φ* (vol%)
HDPE	Al_2_O_3_	15	10, 20, 35, 50
HDPE	Al_2_O_3_	45	10, 20, 35, 50
PP	AlN	5	2, 5, 10, 15, 20, 30, 40, 50
PP	AlN	15	10, 20, 35, 50
PP	Al_2_O_3_	15	10, 30, 50
HDPE	AlN	15	10, 30
TPU	AlN	5	20, 25
TPU	Al_2_O_3_	5	20, 25

**Table 3:** Parameters used in the model to validate the effect of filler–matrix interface and filler orientation.

Thermal Conductivity of Filler, *λ_p_* (W m^−1^ K^−1^)	Thermal Conductivity of Matrix, *λ_m_* (W m^−1^ K^−1^)	Particle Radius, *r*_1_ (µm)	Particles Aspect Ratio, *ξ*	Effect of Orientation $\left\langle \cos^{2}\theta \right\rangle$	Interface Thermal Resistance, *R_int_* (m^2^ K W^−^^1^)	Ref.
200	0.24	2.5	0.05	1/3	1 × 10^−7^	[34]
600	0.14	25	0.1	1/3	23 × 10^−7^	[32]
600	0.14	5	0.095	1/3	23 × 10^−7^	[32]
600	0.24	8	0.2	1	16.6 × 10^−7^	[33]
600	0.24	8	0.2	0	16.6 × 10^−7^	[33]

## Data Availability

Data sharing not applicable.
